# Hydrolysis of untreated lignocellulosic feedstock is independent of S-lignin composition in newly classified anaerobic fungal isolate, *Piromyces* sp. UH3-1

**DOI:** 10.1186/s13068-018-1292-8

**Published:** 2018-10-27

**Authors:** Casey A. Hooker, Ethan T. Hillman, Jonathan C. Overton, Adrian Ortiz-Velez, Makayla Schacht, Abigail Hunnicutt, Nathan S. Mosier, Kevin V. Solomon

**Affiliations:** 1 Department of Agricultural and Biological Engineering, Purdue University, 225 South University Street, West Lafayette, IN 47907-2093 USA; 2 Laboratory of Renewable Resources Engineering (LORRE), Purdue University, 500 Central Drive, West Lafayette, IN 47907-2022 USA; 3 Purdue University Interdisciplinary Life Sciences (PULSe) Program, Purdue University, 155 South Grant Street, West Lafayette, IN 47907-2114 USA; 4 Department of Biological Sciences, Purdue University, 915 West State Street, West Lafayette, IN 47907-2054 USA

**Keywords:** Anaerobic fungi, Neocallimastigomycota, Lignocellulose, Carbohydrate active enzymes, Lignin, Poplar

## Abstract

**Background:**

Plant biomass is an abundant but underused feedstock for bioenergy production due to its complex and variable composition, which resists breakdown into fermentable sugars. These feedstocks, however, are routinely degraded by many uncommercialized microbes such as anaerobic gut fungi. These gut fungi express a broad range of carbohydrate active enzymes and are native to the digestive tracts of ruminants and hindgut fermenters. In this study, we examine gut fungal performance on these substrates as a function of composition, and the ability of this isolate to degrade inhibitory high syringyl lignin-containing forestry residues.

**Results:**

We isolated a novel fungal specimen from a donkey in Independence, Indiana, United States. Phylogenetic analysis of the Internal Transcribed Spacer 1 sequence classified the isolate as a member of the genus *Piromyces* within the phylum Neocallimastigomycota (*Piromyces* sp. UH3-1, strain UH3-1). The isolate penetrates the substrate with an extensive rhizomycelial network and secretes many cellulose-binding enzymes, which are active on various components of lignocellulose. These activities enable the fungus to hydrolyze at least 58% of the glucan and 28% of the available xylan in untreated corn stover within 168 h and support growth on crude agricultural residues, food waste, and energy crops. Importantly, UH3-1 hydrolyzes high syringyl lignin-containing poplar that is inhibitory to many fungi with efficiencies equal to that of low syringyl lignin-containing poplar with no reduction in fungal growth. This behavior is correlated with slight remodeling of the fungal secretome whose composition adapts with substrate to express an enzyme cocktail optimized to degrade the available biomass.

**Conclusions:**

*Piromyces* sp. UH3-1, a newly isolated anaerobic gut fungus, grows on diverse untreated substrates through production of a broad range of carbohydrate active enzymes that are robust to variations in substrate composition. Additionally, UH3-1 and potentially other anaerobic fungi are resistant to inhibitory lignin composition possibly due to changes in enzyme secretion with substrate. Thus, anaerobic fungi are an attractive platform for the production of enzymes that efficiently use mixed feedstocks of variable composition for second generation biofuels. More importantly, our work suggests that the study of anaerobic fungi may reveal naturally evolved strategies to circumvent common hydrolytic inhibitors that hinder biomass usage.

**Electronic supplementary material:**

The online version of this article (10.1186/s13068-018-1292-8) contains supplementary material, which is available to authorized users.

## Background

Lignocellulosic material is an inexpensive and abundant source of carbon that remains underexploited for biofuel production due to its complex heteropolymeric structure that hinders release of fermentable sugars by lignocellulolytic enzymes [1]. Available plant biomass for bioenergy is greatly dependent on geographic location and climate variability, leading to large differences in the types and compositions of the potential substrates [2]. More importantly, the biomass composition strongly affects the performance of a given enzyme cocktail [3]. As a result, the enzyme cocktails that are used to hydrolyze these feedstocks are optimized for individual substrates and are not suitable for more economically viable feedstock streams whose composition fluctuates greatly with market availability [4]. As enzyme cost is a significant bottleneck to the development of economical biofuels, enzyme systems that display superior performance on diverse feedstocks would advance the economic viability of bioenergy [5–7].

Current lignocellulolytic enzymes systems are based on well-known fungi such as *Trichoderma reesei* and *Aspergillus* spp. due to their oversecretion of many glycoside hydrolyases (CAZymes), which are active on the glycosidic bonds of lignocellulosic materials [8]. However, these species do not naturally express all the enzymes needed to fully hydrolyze the sugars contained in plant biomass [9]. For example, β-glucosidases in *T. reesei,* an enzyme essential to release the free glucose, form < 1% of all secreted CAZymes [8]. Thus, enzyme cocktails based on *T. reesei* must be supplemented with enzymes from other species for sufficient activity [10]. The need for cocktail supplementation with enzymes from various species greatly increases enzyme production costs due to capital-intensive parallel enzyme production processes [6, 11]. Therefore, a single species enzyme platform would simplify enzyme production and reduce cost.

Degradation of untreated biomass is common in many underexplored environments that may harbor efficient microbial enzymes for biofuels. One example is the rumen and hindgut of large herbivores where grasses, shrubs, and other untreated fiber-rich plant biomass are processed daily by a consortium of microbes including early divergent Neocallimastigomycota (anaerobic fungi) [12]. While anaerobic fungi are known to harness powerful biomass-degrading enzymes, the ability of these enzymes to hydrolyze diverse plant biomass remains poorly characterized [13]. To date, only five specimens in this phylum have been sequenced and studied in any detail [14]. The fungi of Neocallimastigomycota thrive under mild conditions (pH ≈ 7, 39 °C) and possess large arrays of CAZymes that efficiently degrade untreated plant biomass [13, 15]. However, there are little data on the extent of the cellulosic and xylanosic degradation by these enzymes across a range of lignin compositions.

Given the potential for anaerobic fungi to reduce enzyme production costs, we sought to characterize their enzymatic performance as a function of untreated substrate composition. Here, we report the isolation and taxonomic placement of a recently isolated anaerobic gut fungal specimen (*Piromyces* sp. UH3-1) in the Neocallimastigaceae family. We characterize the ability of UH3-1 to degrade and grow on an array of untreated substrates (e.g., corn stover, switchgrass, orange peel, and sorghum) under mild conditions. Additionally, we measure the free sugars released from untreated poplar across a range of lignin compositions to estimate fungal enzyme performance with feedstock composition. This work suggests that anaerobic fungal enzymes are robust for hydrolysis of diverse untreated lignocellulose and are promising new candidates for lignocellulosic enzyme production.

## Methods

### Isolating a novel species of anaerobic gut fungi

We suspended fresh donkey feces in Hungate tubes containing sterile anaerobic medium C supplemented with 15% clarified rumen fluid (150 ml: Bar Diamond Inc., Parma, ID, USA) under 100% CO_2_ headspace [16]. Suspensions of donkey feces were serially diluted 1000-fold and used as a 10% inoculum in Hungate tubes containing 9 ml anaerobic medium C, supplemented with switchgrass as a carbon source (1% w/v) and chloramphenicol (25 µg/ml; Fisher Scientific, Waltham, MA, USA). After inoculation, the cultures were incubated at 39 °C for 72–96 h.

To obtain axenic cultures, we inoculated roll tubes with liquid fungal culture and propagated individual colonies. Roll tubes were prepared by adding agar (2% w/v), glucose (0.45% w/v), and chloramphenicol (25 µg/ml) to anaerobic medium C under 100% CO_2_ headspace [16]. We melted solid sterile media at 98 °C in a water bath and cooled the media to ~ 45–50 °C prior to the addition of chloramphenicol and 1 ml of inoculum from a liquid fungal culture in mid-exponential phase. Upon inoculation, the tubes were transferred to a benchtop and immediately rolled horizontally creating a uniform agar-inoculum completely coating the walls. The tubes were incubated at 39 °C until colonies were visible, typically between 3 and 5 days. Following incubation, we extracted individual colonies from the agar with a sterile needle while under CO_2_ headspace and transferred them to new Hungate tubes containing 9 ml anaerobic medium C, switchgrass, and antibiotics (chloramphenicol [25 µg/ml in 40% ethanol], streptomycin [40 µg/ml], penicillin [50 µg/ml], and kanamycin [25 µg/ml]). After 72–96 h, we used these cultures to inoculate new roll tubes. Colonies were passaged three times to obtain axenic cultures.

### Substrate preparation

Lignocellulosic substrates were dried by placing them in a Fisher Scientific Isotemp convection oven at 45 °C until they reached approximately 10% moisture. Similarly, we collected the food waste (i.e., orange peel), washed it with deionized water, and dried it to approximately 10% moisture. We milled the dry substrates to 20 mesh (~ 0.85 mm) in a rotary mill. Milled substrates were loaded at 1% w/v prior to the addition of medium C [16]. For all soluble carbon sources, substrates were dissolved in anaerobic medium C at 0.5% w/v prior to being aliquoted into individual Hungate tubes under 100% CO_2_ and autoclaved. Non-lignocellulosic substrates included arabinoxylan from beechwood (Megazyme, Bray, Ireland), xylan from beechwood (Crescent Chemical, Islandia, NY, USA), glucose, arabinose, xylose, cellobiose, filter paper, carboxy methyl cellulose (Fisher Scientific, Waltham, MA, USA), Sigmacell Type 50, and Avicel pH 101 (Sigma Aldrich St. Louis, MO, USA). Genetically modified lines of poplar containing varying molar ratios of syringyl and guiacyl lignin were used to assess the response of *Piromyces* sp. UH3-1 to lignin composition [17, 18]. Poplar at approximately 10% moisture was milled to 40 mesh (~ 0.5 mm), and tubes were loaded with 1% w/v substrate. We tested eight different lines of debarked poplar. Two different lines of wild-type poplar were used in this experiment; NM6, which is a global standard, and INRA 717 from which all the modified lines were constructed [17, 19]. While autoclaved, all biomass used in this study is effectively untreated; empirical calculations of the extent of pretreatment or severity factor are 4 orders of magnitude smaller than mild forms of pretreatment (Log *R*_0_ = 2.10) [20]. Similarly, preliminary studies did not demonstrate significant increases in fungal growth rate or total fungal biomass accumulation when unautoclaved corn stover is used as the substrate (Additional file 1: Figure S8). The autoclaved biomass has not been washed to remove any potential fermentation inhibitors, which hinder enzyme activity [21, 22].

### Microscopy

All images of *Piromyces* sp. UH3-1 were collected via confocal microscopy (Nikon Eclipse Ti Microscope and A1-multiphoton imaging system). Mature fungal cultures containing lignocellulosic material were immobilized in 10% polyacrylamide prior to imaging with 4′,6-diamidino-2-phenylindole (DAPI) (Thermofisher, Waltham, MA, USA). Zoospore images were collected using 3-day corn stover cultures, which were placed in Eppendorf tubes and fixed with glutaraldehyde to a 4% final concentration.

### Species classification

An axenic stock culture (described in Isolating a novel species of anaerobic fungi) was used to inoculate 50 ml serum bottles containing medium C with glucose 0.45% w/v, and chloramphenicol (25 µg/ml in 40% ethanol) [16]. These serum bottles incubated at 39 °C for 3–4 days upon which the gDNA was harvested for species classification. Fungal genomic DNA was isolated with the MoBio PowerFecal kit (Carlsbad, CA, USA), yielding sufficient quality genomic DNA (260/280: 1.9 and 260/230: 1.5) at approximately 2 μg DNA per 50 ml culture. PCR (Phusion DNA polymerase, Thermoscientific, Waltham, MA, USA) was used to amplify the internal transcribed spacer 1 (ITS1) and ITS2 regions of the isolated genomic DNA via JB206/205 primers (5′ GGAAGTAAAAGTCGTAACAAGG 3′ and 5′ TCCTCCGCTTATTAATATGC 3′) yielding an expected amplicon of approximately 700–750 base pairs [23]. We also amplified the D1/D2 portion of the 28S rRNA large subunit (LSU) gene with the NL1/NL4 primers (5′ GCATATCAATAAGCGGAGGAAAAG 3′ and 5′ GGTCCGTGTTTCAAGACGG 3′) [24]. DNA was amplified with the following PCR settings for 30 cycles: annealing at 56 °C, elongating for 60 s at 72 °C and melting at 98 °C. All the same conditions were used for the LSU PCR except the annealing temperature was changed to 67 °C [24]. DNA amplification was checked on an agarose gel and imaged with a c600 Azure Biosystems imager. We concentrated these PCR products with the Zymogen DNA Clean and Concentrator (Zymo Research, Irvine, CA, USA) kit prior to sequence submission at GENEWIZ (South Plainfield, NJ, USA). We assembled the forward and reverse sequence reads of the ITS1 and ITS2 region into a single contig by trimming the ends of reads with poor base calls (> 3 Ns in a 20 base window) and assembling reads with 85% overlap over at least 20 bps with the contig assembly feature in GeneStudio bioinformatics package (ver. 2.2.0.0, GeneStudio, Inc., Suwanee, GA, USA). ITS sequences were also validated by cloning PCR products into the pGEM-T easy cloning vector (Promega, Madison, WI) following the manufacturer’s instructions and sequencing three resulting colonies. Phylogenetic reconstruction was performed using MEGA7 (v 7.0.14). Due to the lack of homogeneity in coverage across the ITS1 and ITS2 sequences in gut fungi, only ITS1 and LSU sequences were used [24]. ITS1 and LSU sequences were analyzed with the maximum likelihood method using a Tamura Nei nucleotide substitution model with 1000 bootstrap replications to estimate the confidence in node clustering.

### Growth curve analyses for characterizing the substrate range of *Piromyces* sp. UH3-1

Fungal growth was tracked according to the method introduced by Thedorou et al. [25]. Briefly, Hungate tubes containing anaerobic medium C and untreated substrate were autoclaved prior to assessing growth (Additional file 1: Table S1) [16]. Every substrate was tested at least in triplicate for growth. Additionally, duplicate uninoculated tubes were used as negative controls for each substrate. Specific growth rates were determined by performing a linear regression of a semi-log plot of accumulated pressure (in psig) versus time (in hours). The Microsoft Excel LINEST function was used for each plot to calculate the slope and exponential phase. The data points in the exponential phase that were linearly increasing and had an *R*^2^ of approximately 0.90 or higher (typically between 48 and 120 h) were used to calculate the specific growth rate on each substrate. We prepared fresh media as described above. Lignocellulosic and insoluble substrates were loaded at 1% w/v while soluble substrates were loaded at 0.5% w/v to keep the total mass of fermentable sugars relatively constant. Tubes were inoculated in a random order to prevent systematic bias in inoculum quality. Pressure accumulation was measured with a pressure transducer (APG, Logan, Utah, USA), every 8 h for 7 days. The growth of UH3-1 on wild type and genetically modified lines of poplar was tested to evaluate the effect of lignin composition on fungal growth (Additional file 1: Tables S2, S3) [17, 19]. For all analyses, individual growth rates and total accumulated pressures were calculated. For data normalization to glucose (Fig. 3d), the average accumulated pressure (in psig) across culture (biological) replicates at 168 h for each substrate was divided by the average accumulated pressure of glucose at 168 h for all of the inoculated tubes. The error for these measurements was propagated accordingly. For data normalization to wild-type poplar (Fig. 5a, b), the same procedure was followed for both growth rate and accumulated pressure.

### Isolation of the carbohydrate active enzymes (CAZymes)

We used a pull-down purification protocol similar to the one by Solomon et al. [13] to isolate and concentrate fungal CAZymes. This procedure exploits the cellulose-binding domains of CAZymes to isolate lignocellulose degrading enzymes [13]. Cultures were centrifuged at 12,800×*g* and the supernatant was transferred to a tube containing approximately 0.4% (w/v) Sigmacell type 50. These tubes were incubated overnight at 4 °C with gentle agitation. Tubes were then centrifuged at 12,800×*g* and the supernatant was discarded. 0.1 M pH 7.0 Tris-NaCl buffer was added to the Sigmacell to elute the cellulose-binding enzymes. The elutions were then stored at 4 °C for further analysis. Protein concentrations were determined by the method introduced by Bradford (Fisher Scientific, Waltham, MA, USA) [26].

### SDS-PAGE and zymography analyses for detailed enzyme characterization

Cellulose-binding proteins were separated and visualized on 10% acrylamide gels run for 70 min at 110 V. Gels were then stained with Sypro Ruby Protein Stain (Fisher Scientific, Waltham, MA, USA). These proteins were also tested for activity via zymography with 0.2% w/v carboxy methyl cellulose (CMC) or 0.4% w/v pectin added to the resolving portion of a 10% acrylamide gel under non-denaturing conditions. The SDS was removed from the gel with slight modification to the procedure of Tseng et al. [27]. The gels were rinsed with ddH_2_O and placed in 0.1 M pH 7.0 Tris-NaCl (TN) buffer containing 25% (w/v) isopropanol (TNI) buffer. Zymogram gels were incubated for 30 min at 4 °C in TNI buffer with gentle agitation. The TNI buffer was then removed and the gel was rinsed two more times with fresh TNI buffer. The zymograms were then washed with 0.1 M pH 7.0 TN-buffer prior to incubation at 39 °C for substrate hydrolysis. CMC zymograms were incubated for 1 h while pectin zymograms were incubated for 24 h. Zymograms were then stained in 0.1% w/v Congo red stain (Fisher Scientific, Waltham, MA, USA) for 30 min and de-stained with 1 M NaCl until the hydrolysis zones appeared relative to the red background. We fixed the zymograms with 0.1 M acetic acid prior to imaging.

### Sugar reducing assay for xylanase activity

UH3-1 xylanase activity was measured after harvesting the cellulose-binding proteins as discussed above. Briefly, we followed the 96 µl microplate procedure introduced by Xiao et al. [28]. However, we used 0.05 M potassium phosphate buffer (pH 7.0) in place of citrate, and a 2% solution of xylan from beechwood (Crescent Chemical, Islandia, NY, USA) as the substrate. Substrate hydrolysis proceeded for 6 h at 50 °C before the generated reducing sugars were measured at 540 nm on a Synergy Neo plate reader (Biotek, Winooski, VT, USA). All samples were measured in triplicate and normalized by total protein. To determine the extent of non-enzymatic xylan degradation, enzyme-free and protein [bovine serum albumin (BSA), Fisher Scientific, Waltham, MA, USA] controls were tested.

### Analyzing the composition of lignocellulosic material after fungal growth

To test the effect of syringyl lignin composition on sugar consumption by *Piromyces* sp. UH3-1, we grew the isolate in 100 ml serum bottles with 50 ml working volume and 1.4% (w/v) solids loading to generate sufficient spent biomass for analysis. Three different poplar constructs were used: 0998-45 (5% S-lignin), wild-type INRA 717 (64% S-lignin), and F5H-64 (98% S-lignin) [17, 19]. After 7 days, the spent lignocellulose and the associated fungal residues were separated from the fermentation media by centrifuging at 5000 RPM for 5 min. After centrifugation, the liquid phase was decanted and the solids were dried for 5 days at 45 °C. The sugar composition of the spent biomass was determined according to the standard methods (Additional file 1: Table S4) [29–31]. Carbohydrates were determined using HPLC analysis (Waters 1525 Pump, Waters Corporation, Milford, MA, USA) equipped with an Aminex™ HPX-87H column (Bio-Rad, Hercules, CA, USA) maintained at 65 °C. The mobile phase was 5 mM aqueous H_2_SO_4_ at a flow rate of 0.6 ml/min. 50 µl of sample was injected, analyzed using a Waters 2414 Refractive Index detector (Waters Corporation, Milford, MA, USA) and quantified using Empower Pro Software (Waters Corporation, Milford, MA, USA). The differences in glucan and xylan composition between the raw and spent biomass were calculated and one-way ANOVA analyses were performed to evaluate the differences in composition.

## Results

### Isolation of a biomass-degrading anaerobic gut fungus from a donkey

**Fig. 1:**
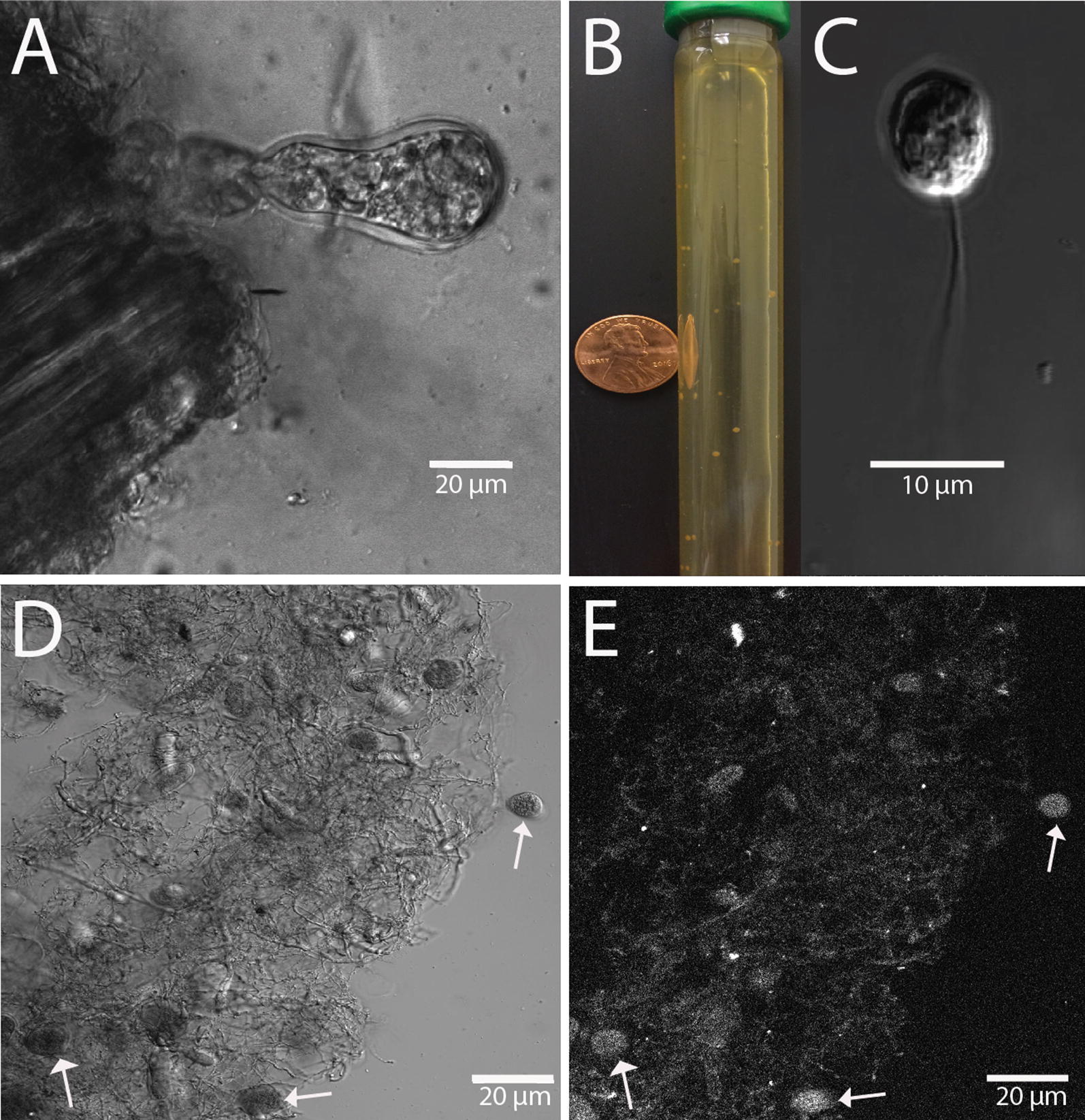
The life cycle of *Piromyces sp.* UH3-1: **A** individual mature sporangia on corn stover (left) displaying ovoid structure. **B** Roll tube used to isolate individual axenic cultures of anaerobic fungi. **C** A uniflagellated zoospore of UH3-1 imaged after zoospore death. **D** Multiple sporangia, demonstrating the predominantly spherical to ovoid structure; arrows indicate individual sporangia in rhizomycelial network. **E** DAPI stain indicating the monocentric nature as zoosporatic nuclei are contained with the sporangia

**Fig. 2:**
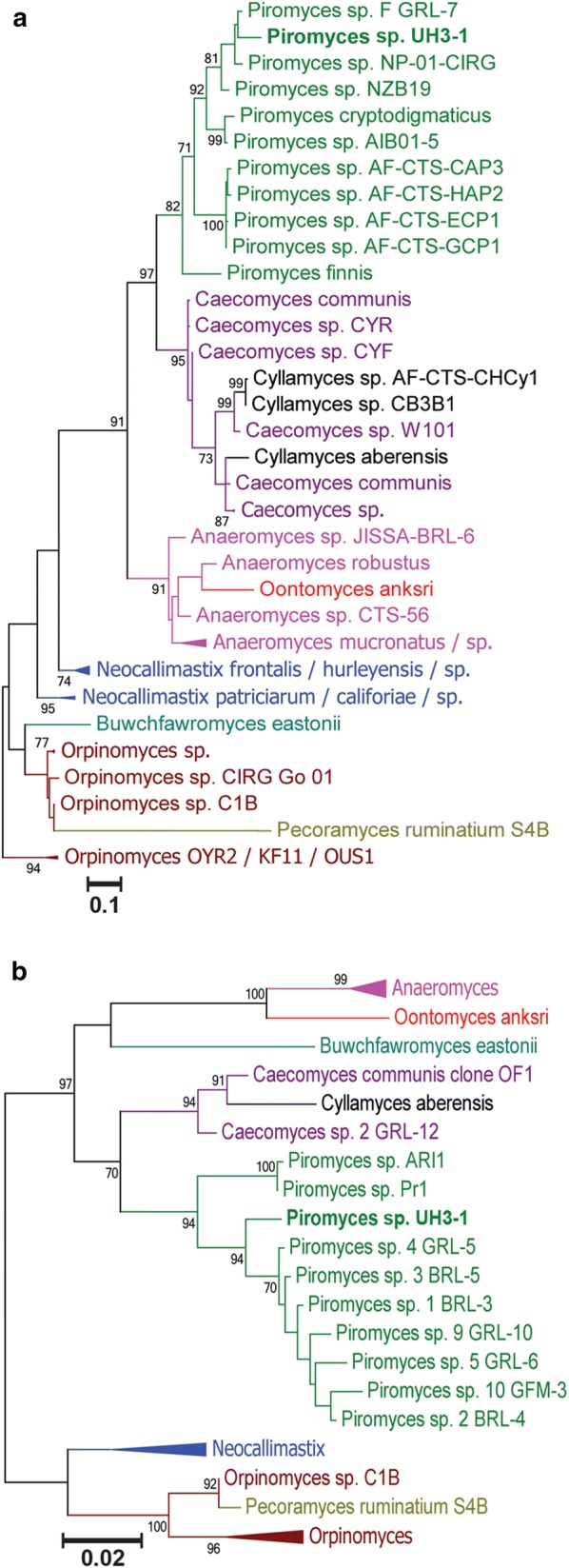
Phylogenetic analyses place our isolate within the genus *Piromyces*: **a** collapsed ITS1 phylogenetic tree and **b** collapsed LSU phylogenetic tree. Fully expanded phylogenetic trees displaying the Genbank accession numbers are available (Additional file 1: Figure S2, S3). Significant bootstrap values from 1000 iterations are indicated to the left of each branch

To identify more robust and efficient CAZymes and microbial systems that may be used for bioenergy applications, we isolated a previously uncharacterized microbe from the fecal samples of a donkey. Light microscopy revealed the presence of non-planktonic microorganisms that grew invasively into the plant substrates, reminiscent of a mature fungal sporangium (Fig. 1A), after 3–4 days of growth with a simultaneous increase in headspace pressure. This isolated organism was cultured to axenic purity by repeated passage through roll tubes containing multiple antibiotics (see “Methods”, Fig. 1B). Further microscopic analysis revealed that this organism produces zoospores with a single flagellum (~ 30 μm long) (Fig. 1C), another key characteristic of the genus *Piromyces* of the fungal phylum Neocallimastigomycota. Additionally, this isolate exhibits endogenous zoosporangial development, where the zoosporangium retains its nuclei. The slow growth, zoospore presence, and the well-differentiated stages of a life cycle (Fig. 1C–E) suggested a fungal specimen. DAPI staining of the nucleic acid in the developing fungal sporangium revealed that this isolate was monocentric (has nuclei only within the zoosporangium) (Fig. 1E), which is also consistent with the morphology of the fungal genus *Piromyces*. Taxonomic classification of our novel fungal isolate was confirmed via phylogenetic analysis [24, 32]. Amplification of the 16s rRNA genes failed, while amplification of the ITS1, ITS2, and the 28s rRNA large ribosomal subunit (LSU) was all successful (Additional file 1: Figure S1) [33]. Therefore, the isolate was definitively fungal in origin, rather than bacterial or archaeal, which agrees with our morphological assessment. We aligned these amplicons against 51 Genbank-deposited anaerobic fungal sequences (division Neocallimastigomycota) and confirmed that our isolate formed a distinct branch within the fungal *Piromyces* genus (Fig. 2) [24, 34]. The monocentric thallus and uniflagellated zoospore, both characteristics of *Piromyces* fungi, further support this placement (Fig. 1) [35]. This organism represents a novel cultured isolate as it has < 90% BLAST similarity to known cultured specimens of anaerobic fungi. Therefore, we classify this organism as *Piromyces* sp. UH3-1.

### Anaerobic fungi degrade and metabolize complex substrates at rates comparable to glucose

**Fig. 3:**
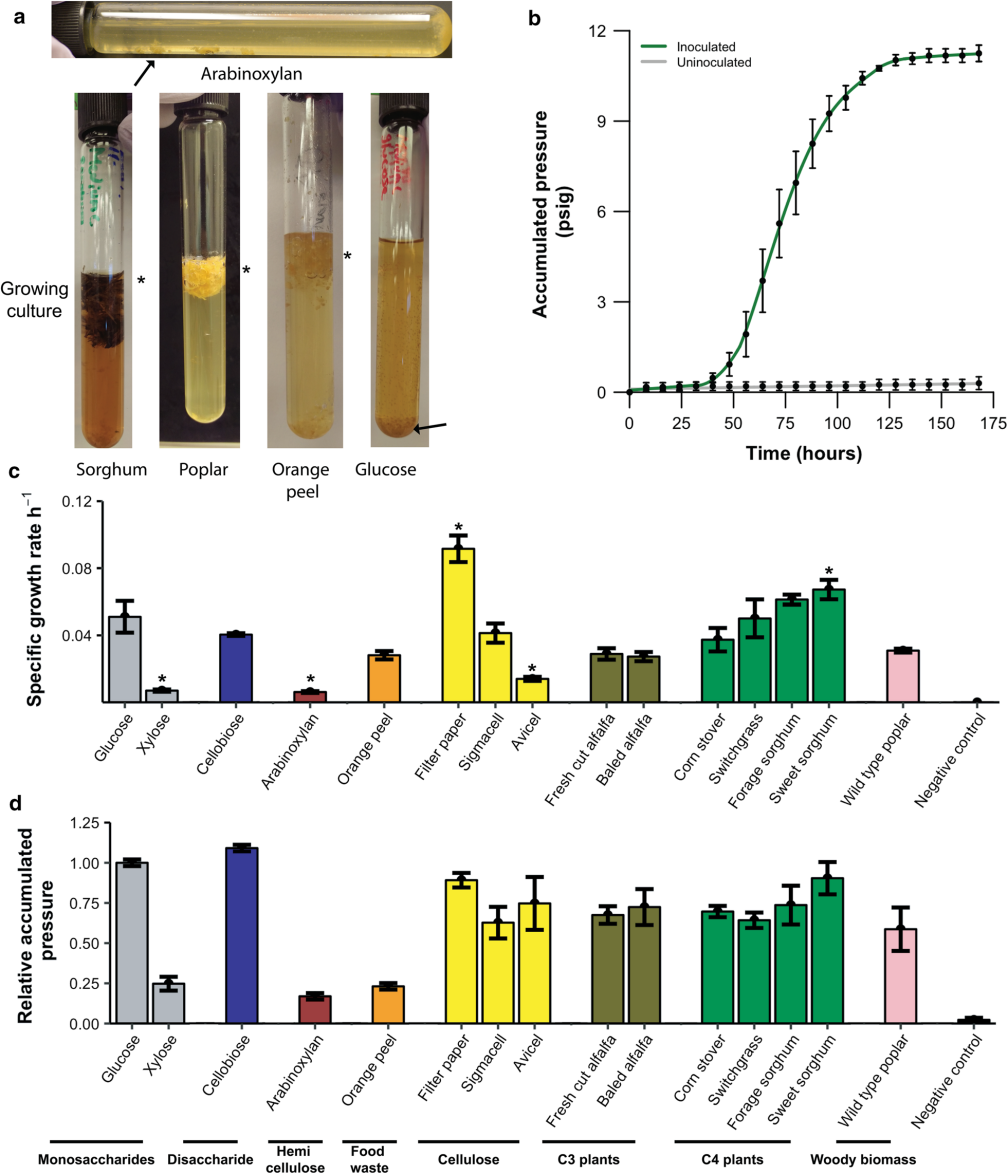
*Piromyces* sp. UH3-1 grows on diverse feedstocks: **a** growth of UH3-1 on soluble substrates leads to colony formation on the walls of the tubes (arrows indicating colony formation). Fungal cultures growing on lignocellulosic substrates float up during fermentation. **b** A representative growth curve of UH3-1 on corn stover. **c**, **d** *Piromyces* sp. UH3-1 degrade and proliferate on a wide array of untreated agricultural wastes, bioenergy feedstocks, and forestry wastes. All accumulated pressures are normalized to glucose. Asterisks denote statistically significant differences in specific growth rate relative to glucose (*p* < 0.05, unpaired *t* test)

Untreated lignocellulosic substrates are rich in sugars that can sustain fungal growth; however, the degradation rate of these substrates into free sugars is frequently limiting for growth. Thus, to estimate hydrolysis efficiency, we assessed the ability of UH3-1 to grow on agricultural residues, bioenergy crops, food wastes, and forestry products that had not undergone pretreatment (Additional file 1: Tables S1, S2). Anaerobic fungi secrete an array of CAZymes that breakdown diverse lignocellulosic material into fermentable sugars that the fungus metabolizes into CO_2_, H_2_, and other fermentation products such as formate, lactate, acetate, and ethanol [36]. Anaerobic gut fungi grow invasively into plant substrates forming rafts of lignocellulose and associated fungal biomass that trap the fermentation gasses. Over time, the rafts capture enough of the fermentation gasses to float just below the surface of the culture medium (Fig. 3a). However, when grown on soluble substrates, the fungi usually adhere to the sides and or bottom of the culture vessel forming biofilms (Fig. 3a). Gas accumulation is proportional to fungal biomass production and may be used as a convenient indicator of growth (Fig. 3b) [25]. Both pressure accumulation and visual analysis were used to assess the ability of UH3-1 to grow on these feedstocks. While the growth rates for these substrates varied significantly, the total pressure accumulations were comparable for lignocellulosic substrates (Fig. 3c, d). Therefore, these results suggest that UH3-1 secretes an array of CAZymes that liberate sufficient sugars, regardless of feedstock composition, to sustain fungal growth into stationary phase.

To determine whether biomass hydrolysis was efficient or limiting for growth, we first established a baseline for growth on simple sugars. Glucose led to vigorous growth (Fig. 3, Additional file 1: Figure S4) and was used as a baseline to which all other substrates were compared. Similarly, the disaccharide cellobiose led to strong fungal growth, suggesting that anaerobic fungi readily produce β-glucosidases that can cleave cellobiose to glucose at a rate in excess of glucose uptake and metabolism (Fig. 3, Additional file 1: Figure S4). In contrast, fungal growth on hemicellulosic components such as xylose (Fig. 3, Additional file 1: Figure S4) led to inconsistent pressure accumulation and a significantly reduced growth rate relative to glucose (*p* = 0.0147, unpaired *t* test). Nonetheless, accumulation of fungal biomass on xylose was consistently observed (Additional file 1: Figure S5). Thus, xylose transport and incorporation into central metabolism likely occur more slowly than six carbon sugars and may be limiting for growth. Taken together, these results suggest that UH3-1 grows primarily on hexose sugars and has robust β-glucosidase activity that is not a bottleneck for biomass hydrolysis, unlike *T. reesei* [37].

While fungal growth on hemicellulose components is poor, it must still remove hemicellulose and other carbohydrate polymers to access the glucose-rich cellulosic portions of lignocellulose. Arabinoxylan, a form of hemicellulose, contains fermentable arabinose and xylose sugars, and is highly abundant in the cell walls of cereals and grasses used as bioenergy crops [38]. Similarly, pectin is a complex and variable component in the middle lamella between the plant cell walls. As this surrounds the energy-rich cellulosic and hemicellulosic polymers, pectin removal or deconstruction is advantageous for efficient lignocellulose hydrolysis [39–41]. The growth of UH3-1 on wheat arabinoxylan and pectin-rich feedstocks such as orange peel, while consistent, was unlike typical microbial growth and non-sigmoidal in nature (Fig. 3, Additional file 1: Figure S4). Thus, their degradation products are unlikely to sustain robust growth. Given the poor growth on these polymeric substrates, we directly analyzed their hydrolysis by collecting the fungal secretome and testing for CAZyme activity.

**Fig. 4:**
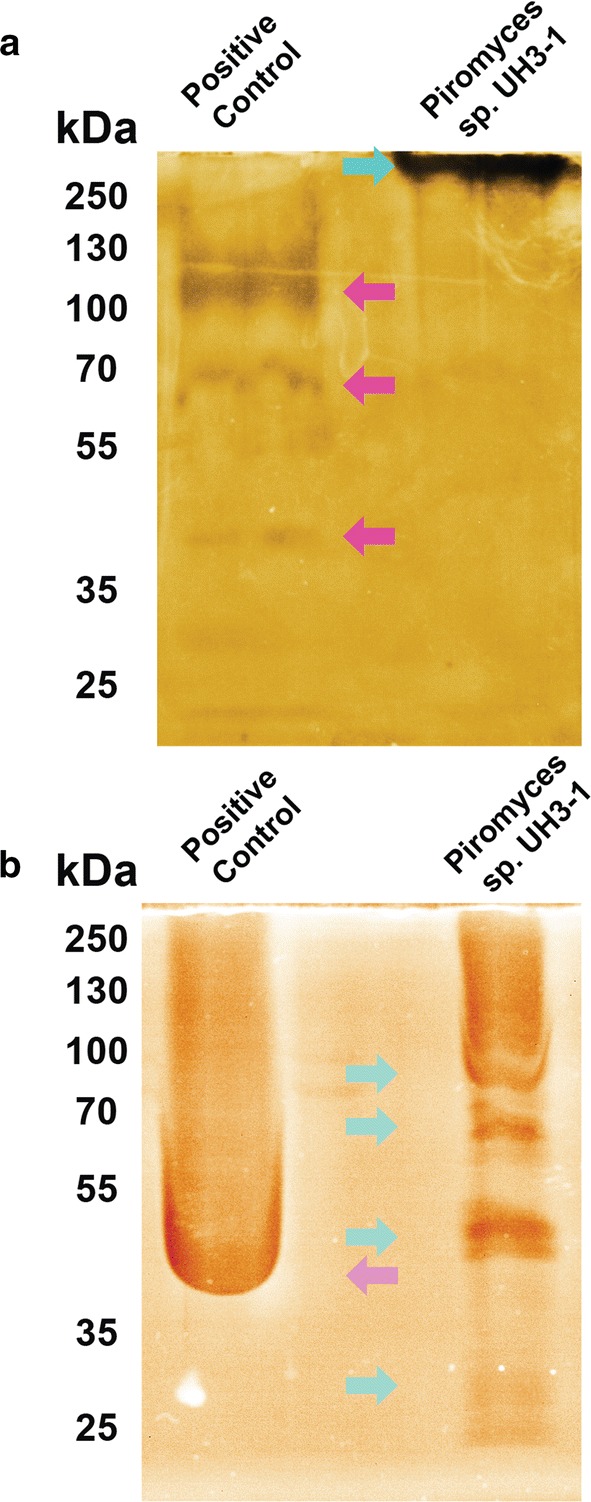
*Piromyces* sp. UH3-1 secretes diverse CAZymes for degrading the polymers of lignocellulose: **a** a pectin zymogram shows strong pectinolytic activity for UH3-1 at the top of the gel (teal arrow), while *Aspergillus* (Viscozyme, positive control) shows multiple bands having pectinolytic activity (pink arrows). **b** A carboxy methyl cellulose zymogram shows distinct cellulolytic activity for multiple proteins of UH3-1 (teal arrows), while *Aspergillus* (Viscozyme, positive control) shows high cellulolytic activity (pink arrow). Controls and experimental samples were loaded with the same total protein mass as measured by a Bradford assay

By isolating the fungal enzymes, we were able to test their activity via zymography, which exploits the ability of some stains to preferentially bind to polysaccharides [42]. Differential staining around individual protein bands results from the consumption of substrate and is positive for hydrolytic activity. Pectin zymograms show a high molecular weight hydrolysis zone indicating that UH3-1 can degrade this complex polymer (Fig. 4a). Similarly, reducing sugar assays reveal strong xylanolytic activity from anaerobic fungal secreted proteins (Additional file 1: Figure S6). Thus, while UH3-1 is unable to efficiently metabolize these substrates, it still expresses an array of CAZymes that breakdown the pectin and hemicellulose components of lignocellulose under mild conditions.

Readily degrading cellulose is critical to efficiently producing energy from renewable plant biomass [43, 44]. Given the variability in cellulose structure between plant sources and preprocessing before enzymatic hydrolysis occurs (e.g., degree of crystallinity, porosity, and specific surface area), we evaluated the efficiency of cellulose hydrolysis by testing three different substrates, which all yielded robust fungal growth (Fig. 3, Additional file 1: Figure S4) [45–47]. Sigmacell from cotton linters and filter paper yielded growth rates that were equal to or in excess of growth on glucose suggesting that cellulase activity is not limiting for growth on lower crystallinity substrates. In contrast, growth on Avicel, a highly crystalline cellulose produced by acid hydrolysis of wood pulp, was reduced by 65% (*p* = 0.0268, unpaired *t* test), likely due to inhibition from the high crystallinity and reduced surface area caused by settling and packing of the substrate in these stationary fermentations [45]. Counterintuitively, growth on filter paper was faster than on glucose (*p* = 0.0023, unpaired *t* test). Despite these differences in growth rate, the total accumulated pressures were comparable, suggesting similar levels of carbon use and thus sugar release, by the fungus independent of substrate crystallinity (Fig. 3d). We sought to further characterize these cellulases by testing their activity through zymography (Fig. 4b). Through this analysis, we identified multiple cellulose-binding proteins having cellulolytic activity. Taken together, these results suggest that UH3-1 efficiently degrades cellulose by expressing multiple cellulases that have high activity in excess of glucose uptake and metabolism.

UH3-1 robustly grew on untreated lignocellulosic feedstocks, regardless of composition or photosynthetic type (Fig. 3). Photosynthetic type (C3 or C4) leads to significant differences in cell wall structure and thus the CAZymes needed to degrade the lignocellulose [48]. For C3 plants, we tested untreated alfalfa (*Medicago sativa)*, which resulted in strong fungal growth (Fig. 3, Additional file 1: Figure S4). Commonly available C4 feedstocks for biofuel production such as corn stover (*Zea mays*), switchgrass (*Panicum virgatum*), and sorghum (*Sorghum bicolor*) were consistently degraded by UH3-1 (Fig. 3b–d, Additional file 1: Figures S4, S7). Several varieties of sorghum, with differing cell wall compositions, were tested as they thrive in different climates and are planted in specific regions, unlike the other tested C4 feedstocks [49–51]. Notably, sweet sorghum was the only lignocellulosic substrate that yielded a significantly higher growth rate when compared to glucose (*p* = 0.0212, unpaired *t* test), possibly due to the excess free sugars common in sweet sorghum [49]. Thus, these results suggest that cell wall composition of untreated lignocellulose does not significantly reduce fungal growth rate, implying that the CAZymes of UH3-1 efficiently degrade these substrates.

### Anaerobic fungal hydrolytic enzymes are robust to lignin composition

Woody biomass such as poplar has been proposed as a feedstock for second-generation biofuel production as it is a fast-growing tree species capable of thriving in diverse geographic locations, and has high biomass yields and high glucan content (> 40%) relative to other commonly used feedstocks (Additional file 1: Tables S2, S3) [43, 52–55]. Furthermore, poplar can be grown on land that is marginally productive for most agricultural crops [56]. However, the lignin in poplar that has not undergone pretreatment is known to strongly affect cellulase and hemicellulase activity [57]. Despite this, UH3-1 still showed strong growth on wild-type poplar (Fig. 3, Additional file 1: Figure S7). This result is consistent with the published data as anaerobic fungi are known to degrade untreated woody biomass [58]. However, as lignin composition may change for diverse feedstocks, we tested fungal growth on transgenic lines of poplar containing varying ratios (5–98%) of syringyl (S)-lignin (Additional file 1: Tables S2, S3, Figure S7) [17, 19]. S-lignin content is known to reduce the growth of some fungi by as much as 80% [59].

**Fig. 5:**
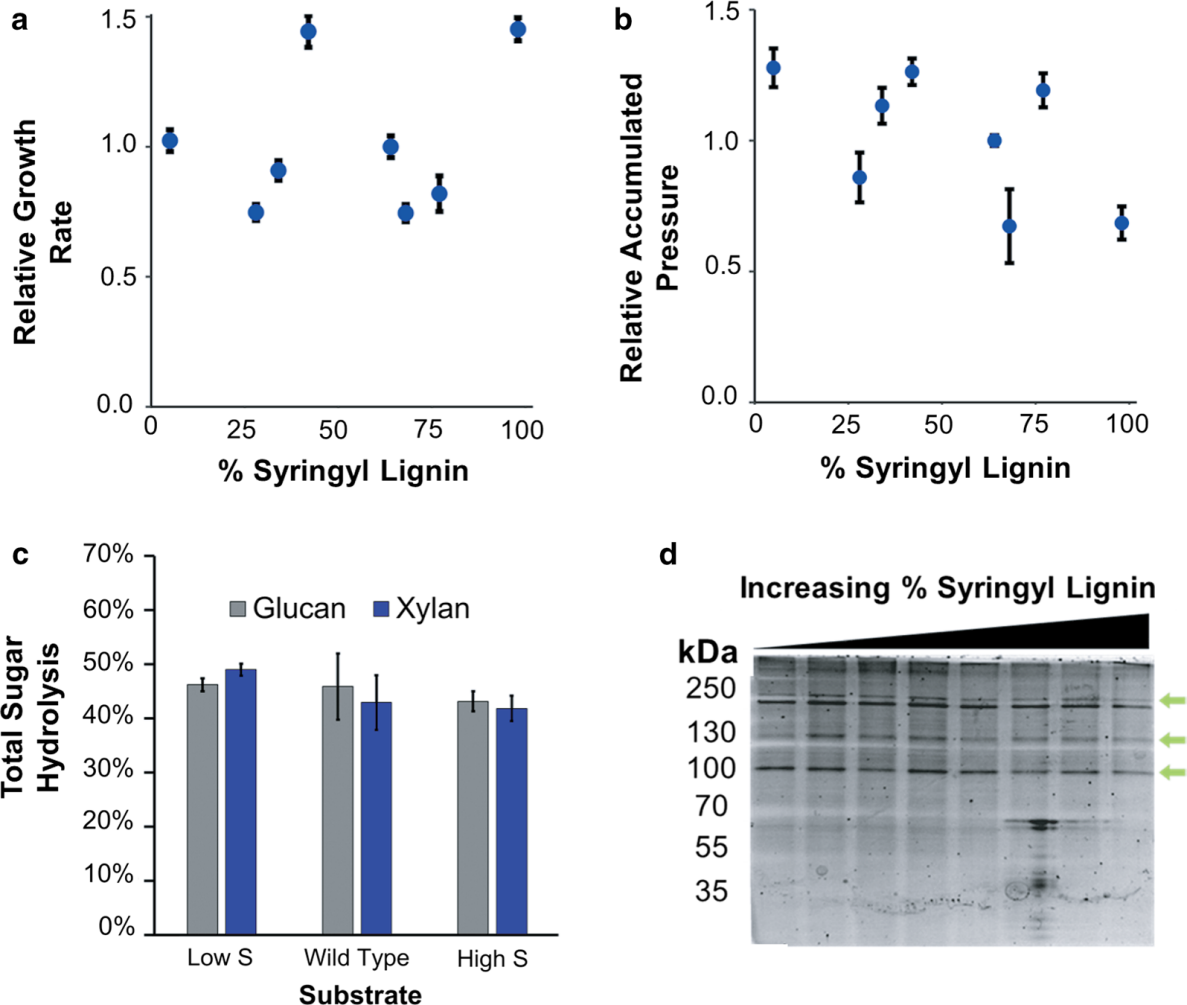
Anaerobic fungal growth and sugar degradation is robust against lignin composition with optimal enzyme expression: **a** relative growth rates of UH3-1 on genetically modified lines of poplar relative to wild-type INRA 717 (64% S-lignin), (*p* = 0.0317, *R*^2^ = 0.1715). **b** Relative fungal biomass accumulations of UH3-1 on genetically modified lines of poplar relative to wild-type INRA 717 (*p* = 0.0011, *R*^2^ = 0.2991). **c** Minimum hydrolysis percentages on three of the lines of poplar [17, 19]. **d** The carbohydrate binding portion of the fungal secretome shows changes in response to S-lignin composition (green arrows)

Our fungal isolate was insensitive to S-lignin content and degraded both S-lignin rich and poor substrates with high efficiency. Both growth rate and fungal biomass accumulation appeared to be independent of S-lignin content. While an ANOVA analysis of these data yielded statistically significant trends for relative growth rate (*p* = 0.0317), and for relative fungal biomass accumulation (*p* = 0.0011), this correlation was weak with *R*^2^ values of 0.1715 and 0.2991, respectively. To further evaluate the degradation of polymeric sugars in the presence of varying S-lignin compositions, we grew UH3-1 on three different poplar constructs and measured the hydrolysis of polymeric sugars to monomeric sugars (Fig. 5c). As fungal rhizomycelia penetrate the plant material, it is currently not possible to distinguish fungal from plant biomass and accurately measure biomass loss, and thus total sugar consumption. However, an analysis of the glucan and xylan contents of spent and fresh poplar biomass, with the conservative assumption that total plant biomass is constant, suggests that UH3-1 metabolizes at least 43% of the glucose sugars, and 42% of the pentose sugars within 168 h (Additional file 1: Table S4). These results are consistent with those reported for an isolate of *Neocallimastix*, a different genera of fungi also within *Neocallimastigomycota* [58]. Specifically, this isolate released glucan and xylan at efficiencies of 47% and 34%, respectively, on untreated poplar after 11 days of growth [58]. Notably, glucan release was independent of S-lignin composition in the poplar constructs tested (wild type vs high S-lignin, *p* = 0.6499; wild type vs low S-lignin, *p* = 0.9951). There was also no significant difference in glucan release between low S-lignin and high S-lignin constructs (*p* = 0.5945). Similar trends were also observed for xylan release (wild type vs high S, *p* = 0.9105; wild-type vs low S-lignin, *p* = 0.1308; high S vs low S constructs, *p* = 0.0771). Taken together, these results suggest that these anaerobic fungal enzymes are robust against inhibitory syringyl lignin content and hydrolyze glucan and xylan in untreated lignocellulose with similar efficiency regardless of lignin composition.

More importantly, while there are no known mechanisms by which anaerobic fungi can metabolize lignin constituents, our results suggest that fungal pathways may exist to recognize lignin composition to increase S lignin resistance. To analyze this, we collected the cellulose-binding portion of the fungal secretome after growth on modified poplar lines (Fig. 5d). The relative concentration of several proteins changed non-linearly with S-lignin content suggesting a complex response to combat S-lignin recalcitrance. More importantly, while there are no known mechanisms by which anaerobic fungi can metabolize lignin constituents, our results do suggest that fungal pathways exist to recognize lignin composition to regulate secretion of enzymes.

## Discussion

Producing biofuels from lignocellulose that are competitive with current energy technologies requires more efficient use of existing biomass reserves in processes that incorporate multiple feedstocks of variable composition. Increasing the number of potential feedstocks will help to protect second generation platforms from changing production conditions that may result due to inconsistency in plant biomass yield, climate variability, and market volatility. One way to move toward this goal is by pretreating plant biomass, which has traditionally been used to overcome lignin inhibition. While pretreatment helps to mitigate these issues, new waste streams are introduced, toxic inhibitors are released that hinder the growth of the fermenting organism, and higher enzyme loadings are required [44, 60–62]. A more promising strategy is, thus, to identify enzyme platforms that readily degrade diverse untreated lignocellulose and are robust to variations in biomass composition. However, for this to be industrially economical, high fermentable sugar conversions are a necessity. Key challenges include product inhibition of the cellulases and the release of lignin, among other contaminants, that can inactivate the secreted enzymes. Despite these barriers, unengineered anaerobic gut fungi such as UH3-1 show strong conversions on untreated plant biomass (Fig. 5). When grown on milled (~ 0.5 mm), untreated corn stover, UH3-1 converts at least 58 and 28 percent of the available glucan and xylan, respectively (Additional file 1: Table S4). These values are comparable to current commercial enzyme cocktails which release 48 and 30 percent of the available glucan and xylan, respectively, on ball-milled (~ 100 micron) corn stover [63]. While hydrolytic rates were not measured as the secretome of UH3-1 is a crude preparation of lignocellulolytic enzymes and other unrelated proteins that increase over time, similar studies of anaerobic fungi suggest that our observed glucan conversion is not limiting and would improve with increased enzyme loading and/or time [64]. In contrast, conversion with current cocktails saturates at these conversions unless supplemented with additional enzyme functionalities [65, 66]. Given the natural ability of UH3-1 to degrade these diverse untreated feedstocks at comparable efficiencies to engineered enzyme cocktails without additional supplementation, further study of their enzymes may provide next generation solutions to critical issues with lignocellulose recalcitrance.

Engineering fungi for enhanced enzyme production has been a subject of considerable research [9, 67]. Both *Aspergillus* and *Trichoderma* are widely used to produce industrial enzyme cocktails, yet these organisms are not the strains that were originally isolated given their natural deficiencies [68, 69]. For example, *Aspergillus* is known to express low amounts of endoglucanases, which are critical for efficiently degrading cellulose [70]. Additionally, the QM6A strain of *Trichoderma reesei* (previously *T. viride*) that was initially isolated went through multiple rounds of mutagenesis to obtain the hyper-producing, catabolite repression-resistant strain Rut-C30 that is the basis for commercial enzyme production [68]. Similar to the original *Trichoderma* QM6A, fungi of *Neocallimastigomycota* are known to have catabolite repression that directly represses CAZyme expression [13, 68]. Despite the presence of catabolite repression, gut fungi still robustly degrade untreated lignocellulose. Manipulating anaerobic gut fungi through mutagenesis or genome engineering would likely lead to improved conversions and make anaerobic fungal enzymes more competitive with current commercial formulations. Similarly, further analysis of how these fungi alter CAZyme expression for diverse untreated lignocellulose may identify new enzymes optimized for certain classes of feedstocks that could be exploited for efficient bioenergy production. However, full-scale industrial exploitation will also require the development of new technologies to cultivate anaerobic fungi at large scale and may be energetically limited by the inherent anaerobic nature of such processes.

## Conclusions

In this work, we present the isolation, taxonomic placement, and characterization of a recently isolated strain of anaerobic gut fungus. We tested fungal growth on diverse untreated feedstocks to estimate the full range of CAZyme activities, and their ability to degrade plant biomass at rates sustainable for fungal growth. UH3-1 thrives on an array of untreated agricultural residues and bioenergy crops by hydrolyzing and fermenting the cellulosic and hemicellulosic fractions of these substrates. Importantly, we show for the first time that anaerobic fungi, such as this isolate, grow and release sugars to similar efficiencies regardless of lignin composition. Thus, this study not only highlights the ability of unengineered gut fungi to degrade diverse untreated lignocellulose, but also suggests that novel adaptations to overcome compositional variability may exist. Characterizing these adaptations and isolating the responsible enzymes may lead to more efficient enzyme cocktails that can more fully use available renewable biomass for lignocellulosic biofuel production.

## Additional file

**Additional file 1: Table S1.** NREL compositional analysis of the renewable plant biomass used in this study. **Table S2.** NREL Compositional analysis of poplar constructs used in this study. **Table S3.** Syringyl lignin content of the poplar constructs used in this study. **Table S4.** Sugar conversion percentages for untreated plant biomass used in this study. **Figure S1.** UH3-1 DNA controls: A) In lane 2, the bacterial V4/V5 primers do not amplify genomic DNA of this organism. However, in lane 3, the JB206/JB205 primers amplify the ITS1 region of this *Piromyces* isolate. B) The NL1/NL4 primers amplify UH3-1 28s rDNA. **Figure S3.** Expanded LSU phylogenetic tree with accession numbers. **Figure S4.** UH3-1 growth curves on various carbon sources. **Figure S5.** UH3-1 shows visible fungal biomass accumulation on media containing xylose. The top tube was inoculated and shows a high amount of fungal biomass, while the bottom tube was used as a negative control, and was not inoculated. **Figure S6.** UH3-1 shows strong xylanolytic activity on xylan from beechwood at 50 °C, pH 7 for six hours of hydrolysis. Values normalized to Viscozyme. **Figure S7.** UH3-1 growth curves on lignocellulosic substrates and the genetically modified lines of poplar used for the S lignin analysis. **Figure S8.** Autoclaving corn stover at 120 °C for 30 min does not significantly enhance fungal growth rate or total accumulated pressure. This autoclaved corn stover was not washed to remove any potential fermentation inhibitors that would be expected to reduce fungal growth. N = 4.

## Abbreviations

ANOVA: analysis of variance
BSA: bovine serum albumin
CAZyme: carbohydrate active enzyme
ITS: internal transcribed spacer
PCR: polymerase chain reaction
SDS-PAGE: sodium dodecyl sulfate polyacrylamide gel electrophoresis
S-Lignin: syringyl lignin
TN: Tris NaCl buffer
TNI: Tris NaCl with isopropanol
DAPI: 4′,6-diamidino-2-phenylindole
LSU: 28S rRNA large ribosomal subunit
