# Supplementary material for: Hydrolysis of untreated lignocellulosic feedstock is independent of S-lignin composition in newly classified anaerobic fungal isolate, Piromyces sp. UH3-1
Source: Biotechnol Biofuels. 2018 Oct 27;11:293. doi: 10.1186/s13068-018-1292-8 (PMC6203967; doi:10.1186/s13068-018-1292-8)
Supplement: Supplementary file 1 — Additional file 1: Table S1. NREL compositional analysis of the renewable plant biomass used in this study. Table S2. NREL Compositional analysis of poplar constructs used in this study. Table S3. Syringyl lignin content of the poplar constructs used in this study. Table S4. Sugar conversion percentages for untreated plant biomass used in this study. Figure S1. UH3-1 DNA controls: A) In lane 2, the bacterial V4/V5 primers do not amplify genomic DNA of this organism. However, in lane 3, the JB206/JB205 primers amplify the ITS1 region of this Piromyces isolate. B) The NL1/NL4 primers amplify UH3-1 28s rDNA. Figure S3. Expanded LSU phylogenetic tree with accession numbers. Figure S4. UH3-1 growth curves on various carbon sources. Figure S5. UH3-1 shows visible fungal biomass accumulation on media containing xylose. The top tube was inoculated and shows a high amount of fungal biomass, while the bottom tube was used as a negative control, and was not inoculated. Figure S6. UH3-1 shows strong xylanolytic activity on xylan from beechwood at 50 °C, pH 7 for six hours of hydrolysis. Values normalized to Viscozyme. Figure S7. UH3-1 growth curves on lignocellulosic substrates and the genetically modified lines of poplar used for the S lignin analysis. Figure S8. Autoclaving corn stover at 120 °C for 30 min does not significantly enhance fungal growth rate or total accumulated pressure. This autoclaved corn stover was not washed to remove any potential fermentation inhibitors that would be expected to reduce fungal growth. N = 4. [file 13068_2018_1292_MOESM1_ESM.docx]

Additional file for

**Hydrolysis of untreated lignocellulosic feedstock is independent of S-lignin composition in newly classified anaerobic fungal isolate, *Piromyces* sp. UH3-1**

**Table S1:** NREL compositional analysis of the renewable plant biomass used in this study.


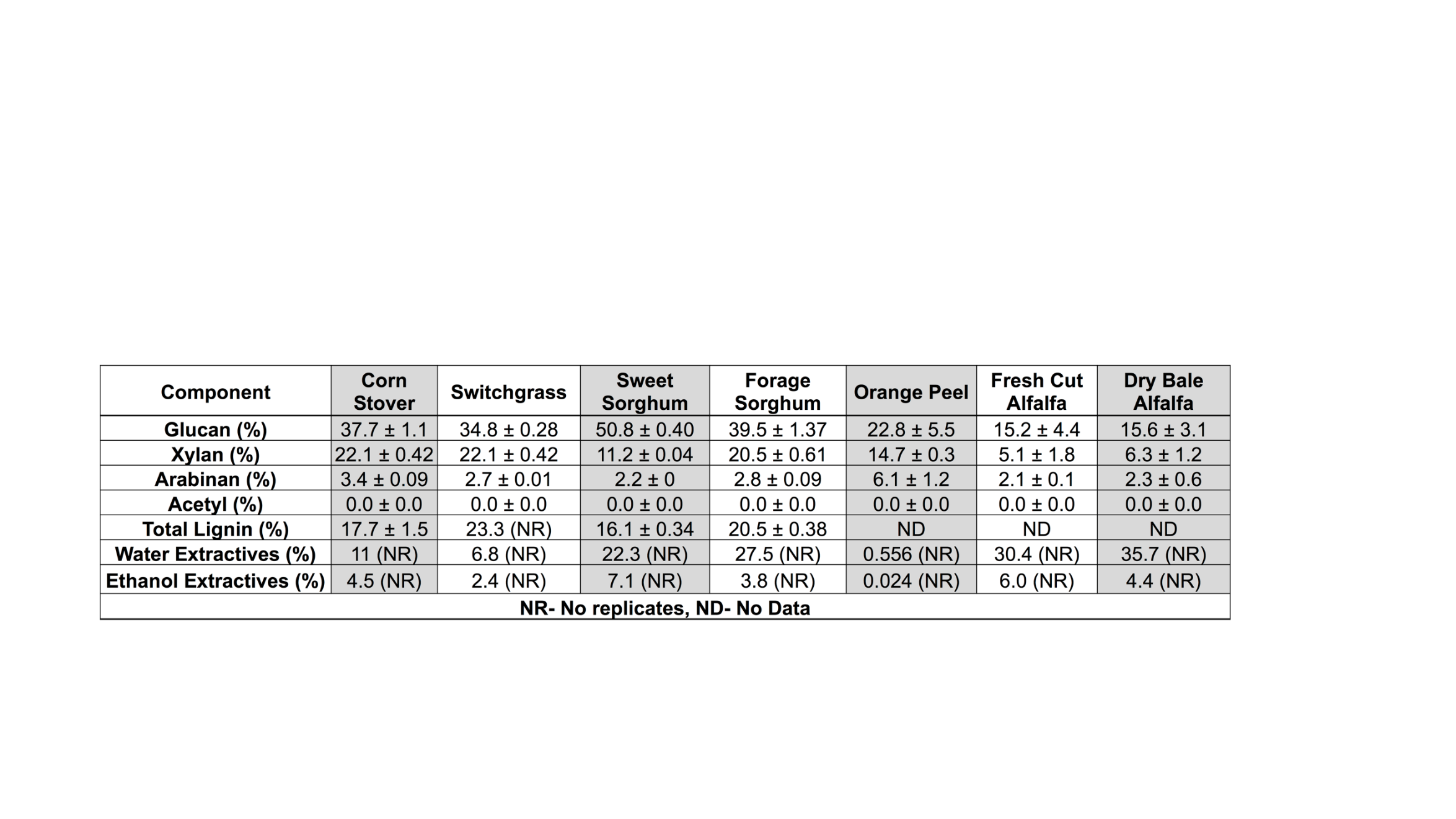


**Table S2:** NREL Compositional analysis of poplar constructs used in this study.
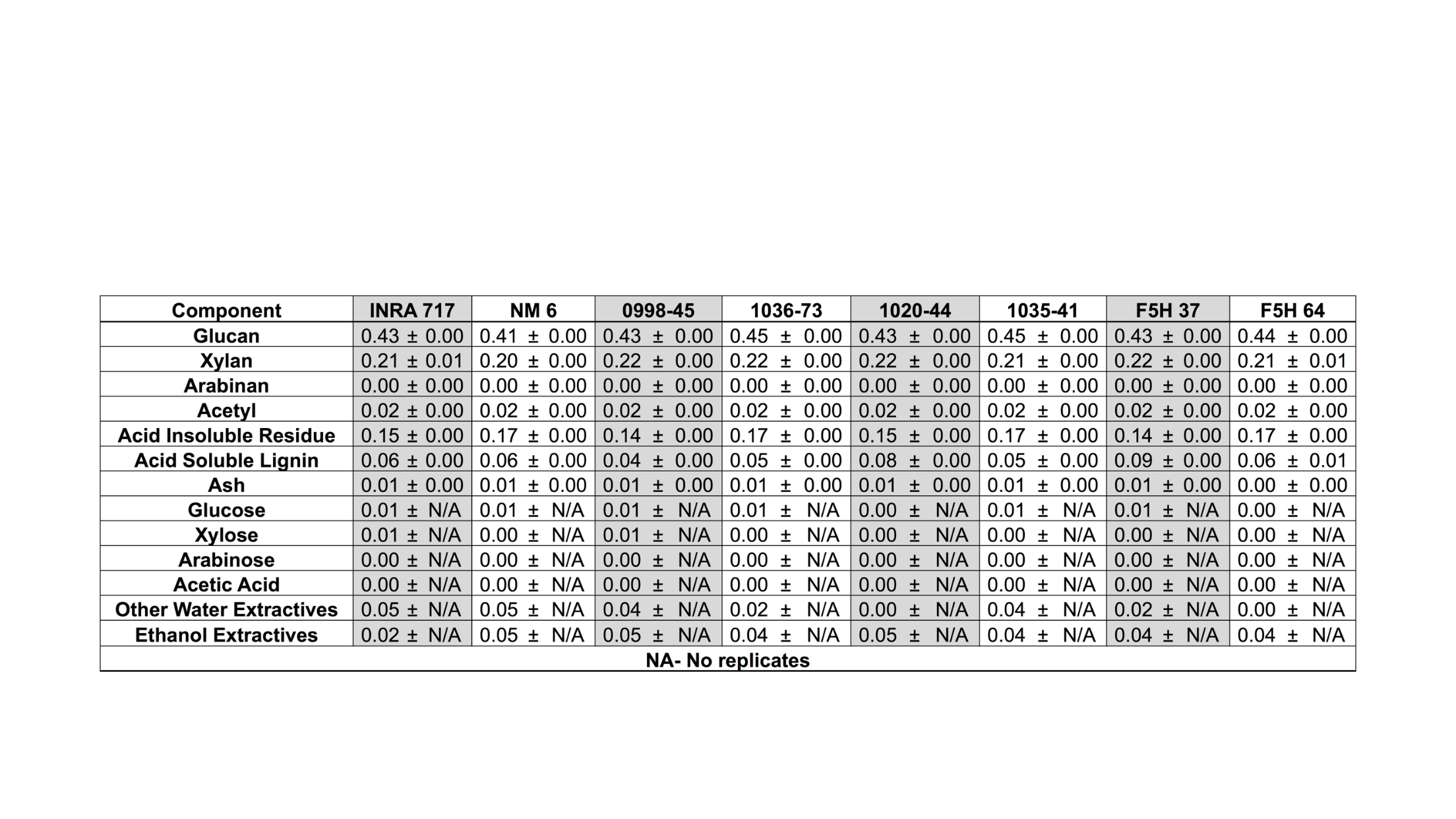


**Table S3:** Syringyl lignin content of the poplar constructs used in this study.

**
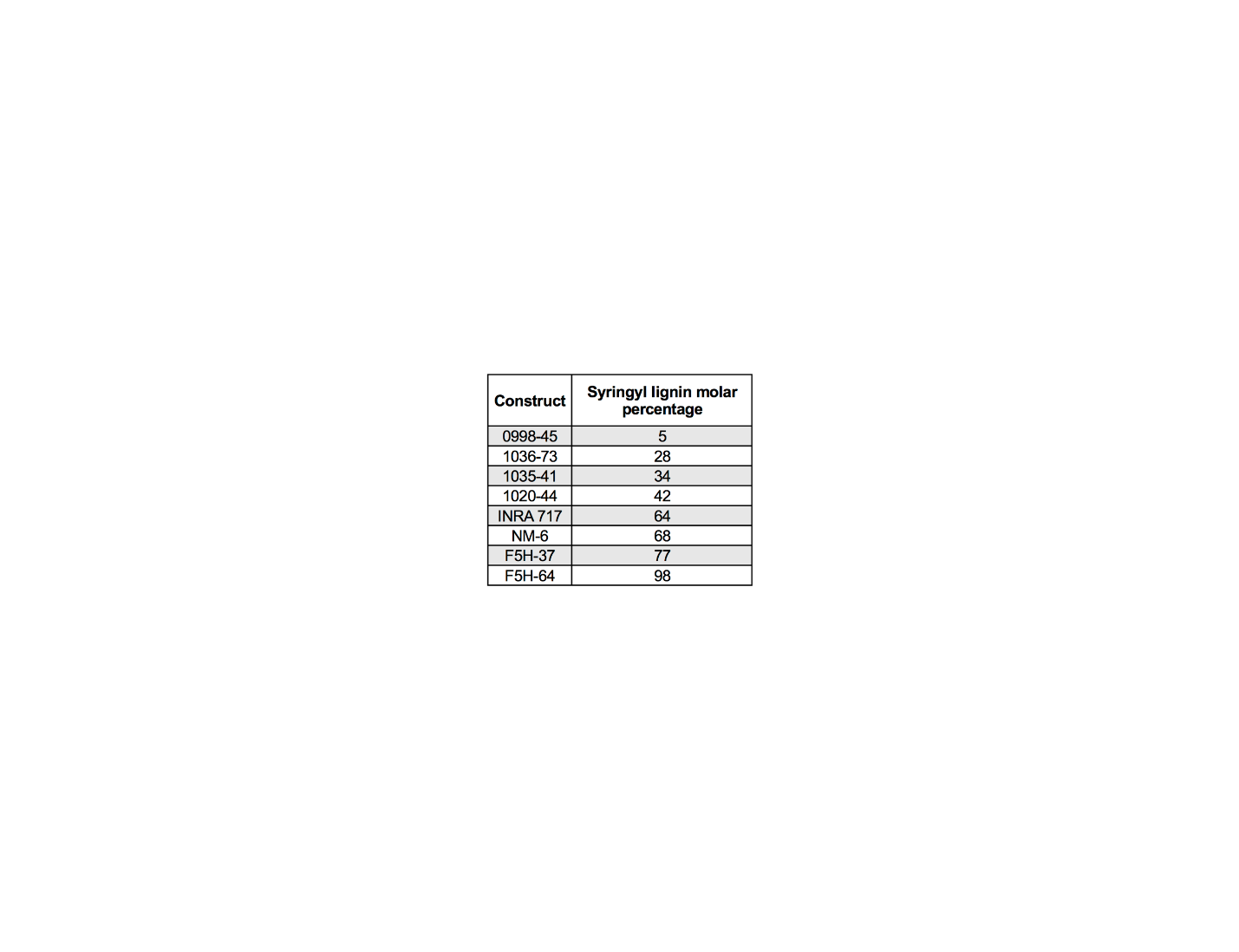
**

**Table S4:** Sugar conversion percentages for untreated plant biomass used in this study.

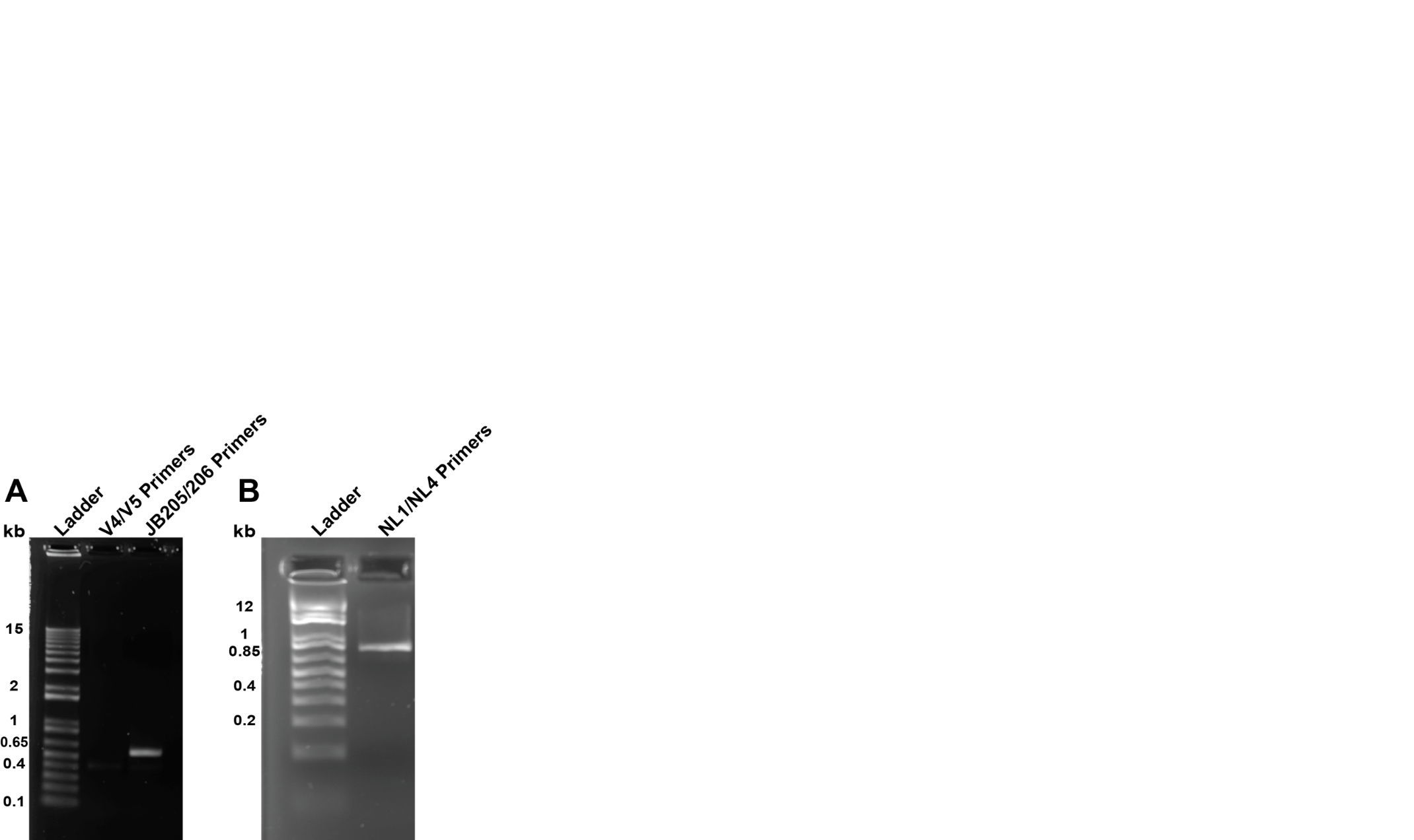


**Figure S1 UH3-1 DNA controls:** A) In lane 2, the bacterial V4/V5 primers do not amplify genomic DNA of this organism. However, in lane 3, the JB206/JB205 primers amplify the ITS1 region of this *Piromyces* isolate. B) The NL1/NL4 primers amplify UH3-1 28s rDNA.


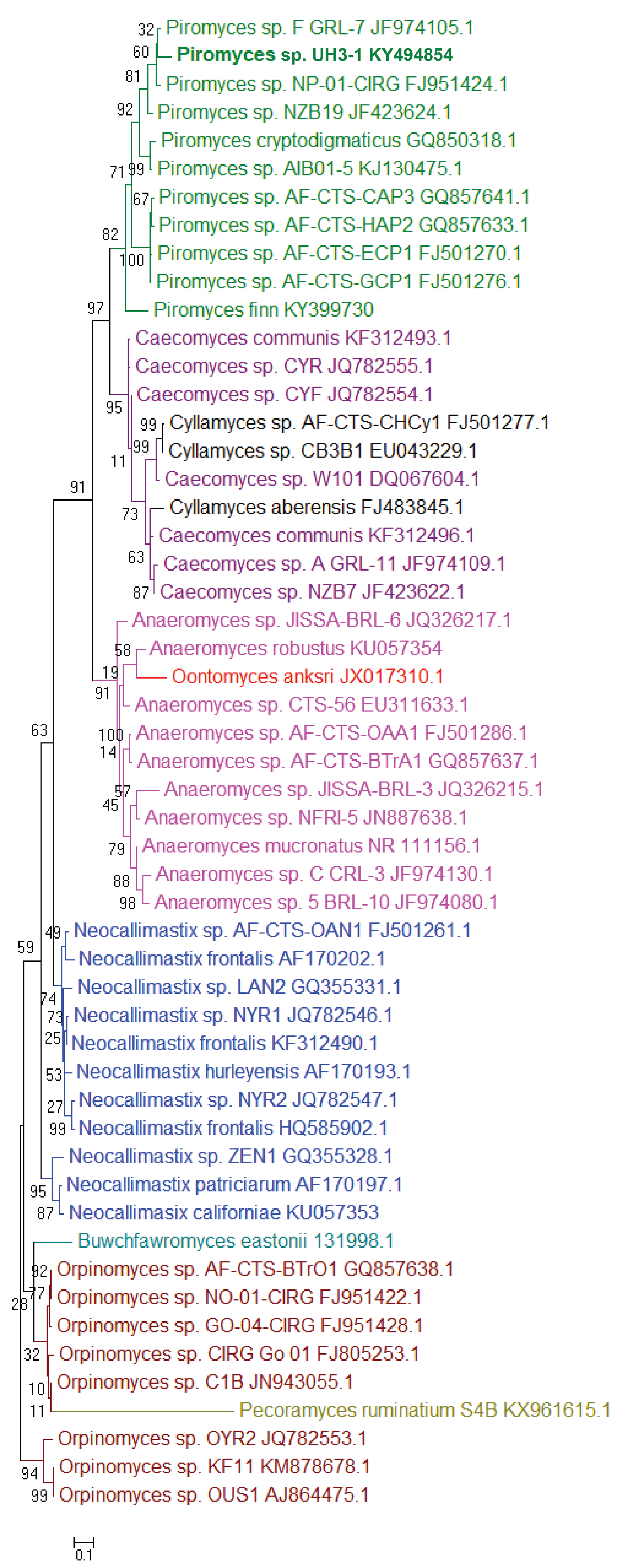


**Figure S2:** Expanded ITS1 phylogenetic tree with accession numbers.


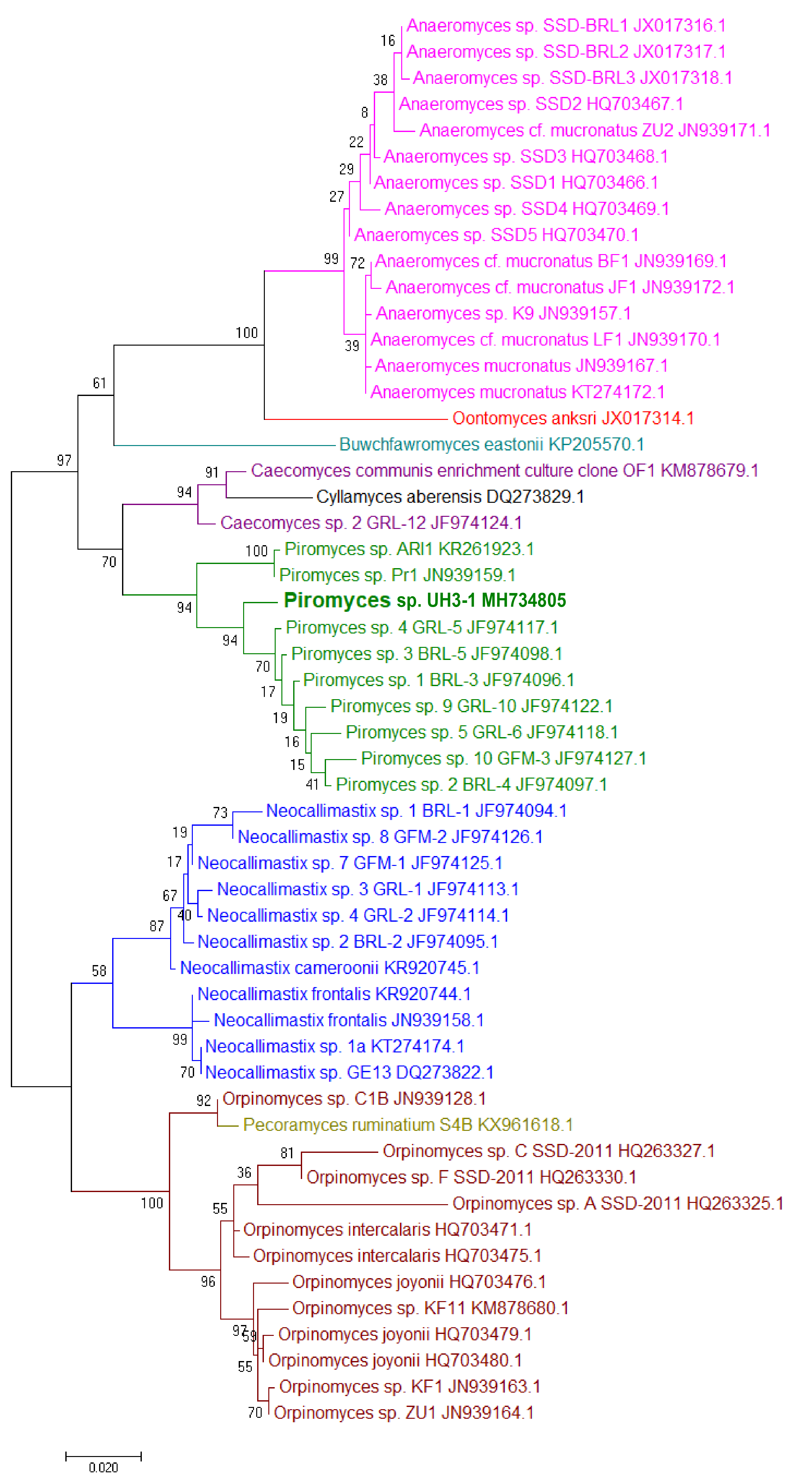


**Figure S3:** Expanded LSU phylogenetic tree with accession numbers


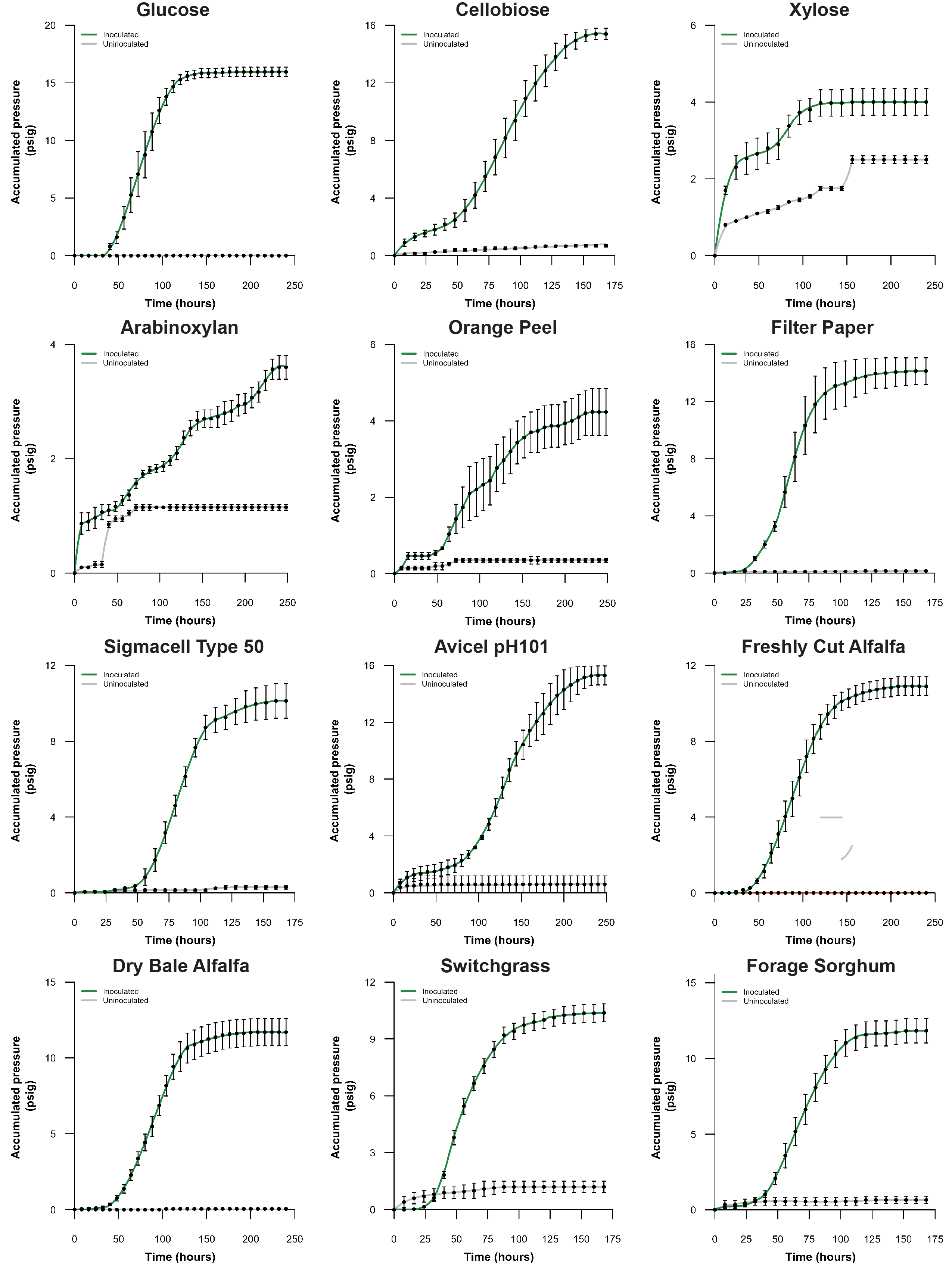


**Figure S4:** UH3-1 growth curves on various carbon sources.

**
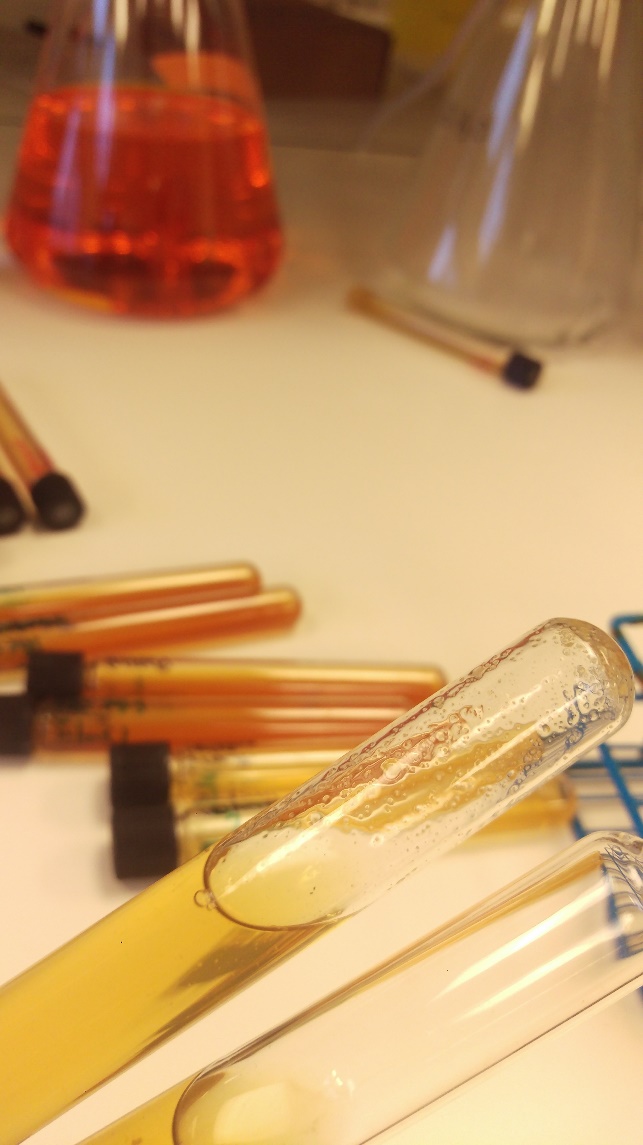
**

**Figure S5:** UH3-1 shows visible fungal biomass accumulation on media containing xylose. The top tube was inoculated and shows a high amount of fungal biomass, while the bottom tube was used as a negative control, and was not inoculated.


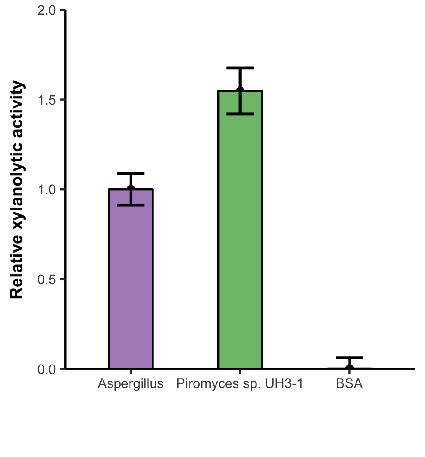


**Figure S6:** UH3-1 shows strong xylanolytic activity on xylan from beechwood at 50° C, pH 7 for six hours of hydrolysis. Values normalized to Viscozyme.


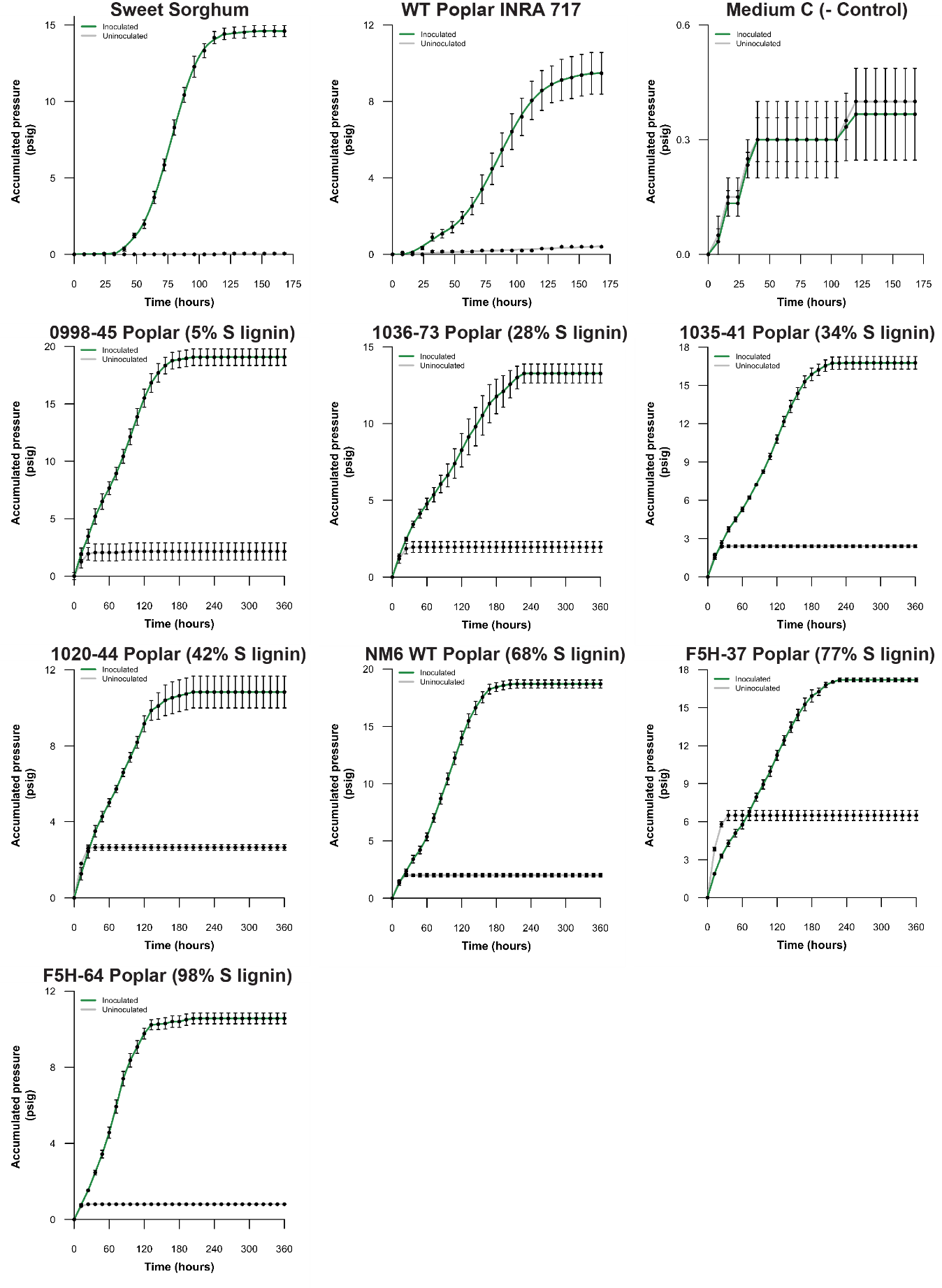


**Figure S7**: UH3-1 growth curves on lignocellulosic substrates and the genetically modified lines of poplar used for the S lignin analysis.


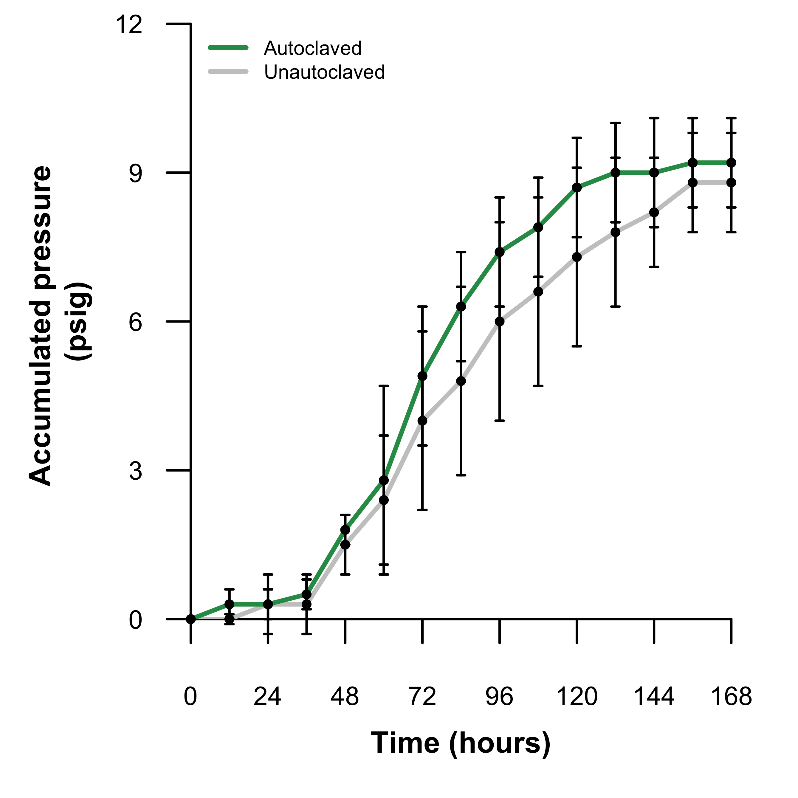


**Figure S8:** Autoclaving corn stover at 120 °C for 30 minutes does not significantly enhance fungal growth rate or total accumulated pressure. This autoclaved corn stover was not washed to remove any potential fermentation inhibitors that would be expected to reduce fungal growth. N=4
